# Underground hydrogen storage: The techno-economic perspective

**DOI:** 10.12688/openreseurope.16974.2

**Published:** 2024-05-29

**Authors:** Eleni Gianni, Pavlos Tyrologou, Nazaré Couto, Júlio Ferreira Carneiro, Eva Scholtzová, Nikolaos Koukouzas

**Affiliations:** 1 Chemical Process & Energy Resources Institute (CPERI), Centre for Research and Technology Hellas (CERTH), Athens, 15125, Greece; 2 CENSE – Center for Environmental and Sustainability Research & CHANGE - Global Change and Sustainability Institute, NOVA University, Lisbon, 2829-516, Portugal; 3 Departamento de Geociências, Escola de Ciências e Tecnologia, Universidade de Evora, Evora, Évora District, 7000-671, Portugal; 4 Institute of Inorganic Chemistry, Slovak Academy of Sciences, Bratislava, 84536, Slovakia

**Keywords:** energy transition, underground hydrogen storage, porous media, rock cavities, technical parameters, economic requirements

## Abstract

The changes in the energy sector after the Paris agreement and the establishment of the Green Deal, pressed the governments to embrace new measures to reduce greenhouse gas emissions. Among them, is the replacement of fossil fuels by renewable energy sources or carbon-neutral alternative means, such as green hydrogen. As the European Commission approved green hydrogen as a clean fuel, the interest in investments and dedicated action plans related to its production and storage has significantly increased. Hydrogen storage is feasible in aboveground infrastructures as well as in underground constructions. Proper geological environments for underground hydrogen storage are porous media and rock cavities. Porous media are classified into depleted hydrocarbon reservoirs and aquifers, while rock cavities are subdivided into hard rock caverns, salt caverns, and abandoned mines. Depending on the storage option, various technological requirements are mandatory, influencing the required capital cost. Although the selection of the optimum storage technology is site depending, the techno-economical appraisal of the available underground storage options featured the porous media as the most economically attractive option. Depleted hydrocarbon reservoirs were of high interest as site characterisation and cavern mining are omitted due to pre-existing infrastructure, followed by aquifers, where hydrogen storage requires a much simpler construction. Research on data analytics and machine learning tools will open avenues for consolidated knowledge of geological storage technologies.

## Introduction – Climate change and mitigation measures

Over the last few years and certainly after the Paris agreement in 2015 ^1^, there has been intense pressure for the energy transition from using fossil fuels to alternative means. To achieve this, policymakers have enacted several tools. To combat climate change, the EU has created the EU Emissions Trading System (EU ETS), a vital tool for reducing greenhouse gas emissions cost-effectively. The EU ETS is a market for CO _2 _trading based on the polluter pays principle and works on the 'cap and trade' basis. A cap is set on the total amount of certain greenhouse gases that industries covered by the system can emit. The cap is reduced over time so that total emissions fall. Within the cap, the industries buy or receive emissions allowances, i.e., trade with one another as needed. The limit on the total number of available allowances provides a value on emissions ^2, 3^.

After each year, an industrial emitter must cover the emissions produced entirely by its allowance or by additional traded ones; otherwise, heavy fines are imposed. If an industrial emitter reduces its emissions, it can either keep the spare allowances to cover its future needs or sell them to another installation short of allowances ^3^. The revenue generated from this process is used to encourage investment in renewable energy and reduce the use of fossil fuels. The idea behind this is to put a price on carbon emissions and make it more expensive to pollute the environment. The goal is to reduce greenhouse gas emissions, mitigate the impacts of climate change, and create a more sustainable economy ^4, 5^.

Until 2020 the CO _2_ trade prices were quite affordable, with transactions between 20–33 dollars per tonne. However, after 2021, there is an increasing trend in the price of emissions, reaching a high peak in August 2022 at 96 euros per tonne. After that, CO _2_ emission trade prices were exchanged between 70 to 98 dollars/tonne and never below 70 dollars per tonne ^6^.

Carbon Capture Utilisation and Storage technologies are rendered sustainable after 40 dollars (euros)/tonne of CO _2_ allowances ^7^. In other words, using carbon capture utilisation and storage (CCUS) is financially profitable rather than trading and buying allowances from the EU ETS.

**Figure 1.**
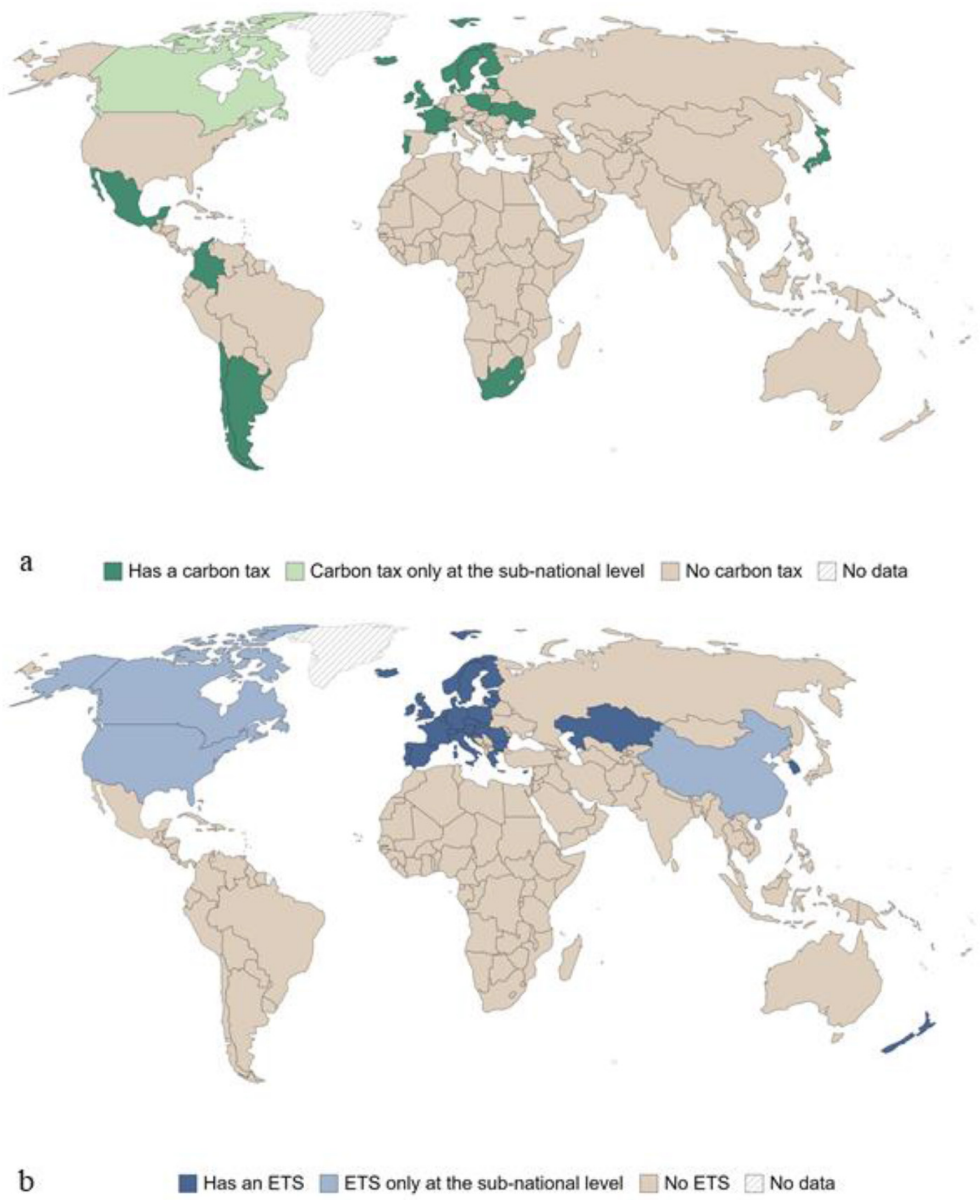
Countries with a carbon tax in 2020, marked if at least one sector has implemented one carbon emissions tax instrument ( **a**), and countries with carbon emissions trading system, marked if at least one sector is covered by one (This figure has been reproduced with permission CC BY-NC-ND 4.0 Attribution-Non-Commercial-No-Derivatives 4.0 International from 2).

Furthermore, many countries that are not within the EU have also adopted similar Carbon tax initiatives ( Figure 1).

The EU's commitment to the Paris Agreement has materialised with the Green Deal ^8^, which introduced measures to reduce greenhouse gas emissions, improve energy efficiency and promote renewable energy use through new technologies development. To achieve the aforementioned, the Green Deal introduced the EU taxonomy, a classification system to identify and provide the framework to promote environmentally sustainable activities.

Within this framework, renewable energy development is socially and financially profitable since it reduces greenhouse gas emissions and mitigates the impacts of climate change. However, renewable energy sources such as wind and solar power are subject to fluctuations in availability, which can impact grid stability, i.e. the ability of an electrical grid to maintain a stable, secure, and reliable supply of electricity.

Grid stability can be achieved by deploying green hydrogen produced by water hydrolysis utilising renewable energy. The produced hydrogen can be stored and used when needed. Green hydrogen is environmentally sustainable since it does not emit greenhouse gases during production or energy conversion. Thus, it can balance the grid by providing additional energy during periods of high demand and reducing (or storing) energy production during periods of low demand.

Since green hydrogen supports the transition to a low-carbon economy and helps reduce greenhouse gas emissions, it is considered a sustainable activity under the EU Taxonomy. As a result, the European Commission has identified green hydrogen as a priority area for investment and has established a dedicated action plan to deploy green hydrogen. Hydrogen storage locations within Europe are of great significance since they provide an energy supply stability in the short and the long term. Furthermore, based on the recent example from the war in Ukraine, they provide an alternative solution for the Green Deal and the energy transition from the traditional fossil fuels and the volatility. Additionally, green hydrogen promotes renewable energy and independence of Europe from alternative sources.

This paper is dedicated to the techno-economic aspects of massive underground green hydrogen storage technologies that will facilitate the wider adoption of hydrogen to mitigate the impacts of climate change and stabilise the grid system. It should be noted that various aspects of the analysis provided below are also applied to other colours/production varieties of hydrogen, such as turquoise, orange, white (natural), or blue. The production cost of each hydrogen colour is given in Table 1. In addition, since the methodology for the underground site selection is similar for all the available technologies, including the geological, geophysical, and geomechanical investigation of an area, their detailed description is omitted in this study, and the reader is directed to a selection of available literature ^8– 10^. However, where specific circumstances exist affecting the aforementioned parameters resulting in significant changes in costs (e.g. brine disposal for salt caverns), a detailed description is given for the proper cost estimation. Afterall, the present study main outputs can be summarised to:

1. Description of technical requirements for each underground geological environment.
2. Economic analysis of the available technologies for underground hydrogen storage.
3. Prioritisation of the most affordable technologies based on the general techno-economic description without taking into consideration the site-specific nature of the economic comparison.

**Table 1.** Production cost of hydrogen varieties ^ii^.

Hydrogen colour	Production cost ($/kg)
Black/Brown	1.18 – 2.36 ^1^
Gray	1.07
Green	2.14
Blue	1.61 – 2.68 ^2^
Turquoise	Aiming to the same price as gray
Pink	2.14 – 6.43 ^3^
White	Produced naturally

The given costs are from 2022 transferred to on-date values based on inflation rates for the dollar that is based on the latest US government Consumer Price Index data. ^1^ Depending on coal price. ^2^ Depending on the natural gas input cost. ^3^ Depending on the production system in place. Data collected from 11.

## State of the art

As discussed above, the use of green hydrogen and hydrogen storage can contribute to mitigating climate change by reducing greenhouse gas emissions. It can also provide a means of decarbonising difficult-to-electrify sectors. However, developing and implementing green hydrogen and hydrogen storage infrastructure requires significant investment and coordination. Both above ground facilities, such as surface pipelines, pressure tanks, metal hydride storage, chemical storage, and hydrogen carriers, and subsurface facilities are under development for hydrogen storage ^12, 13^. Above ground facilities are independent of geological conditions and geological research can be avoided, however they are more expensive in terms of levelized cost of storage when compared with underground storage technologies ^14^.

**Figure 2.**
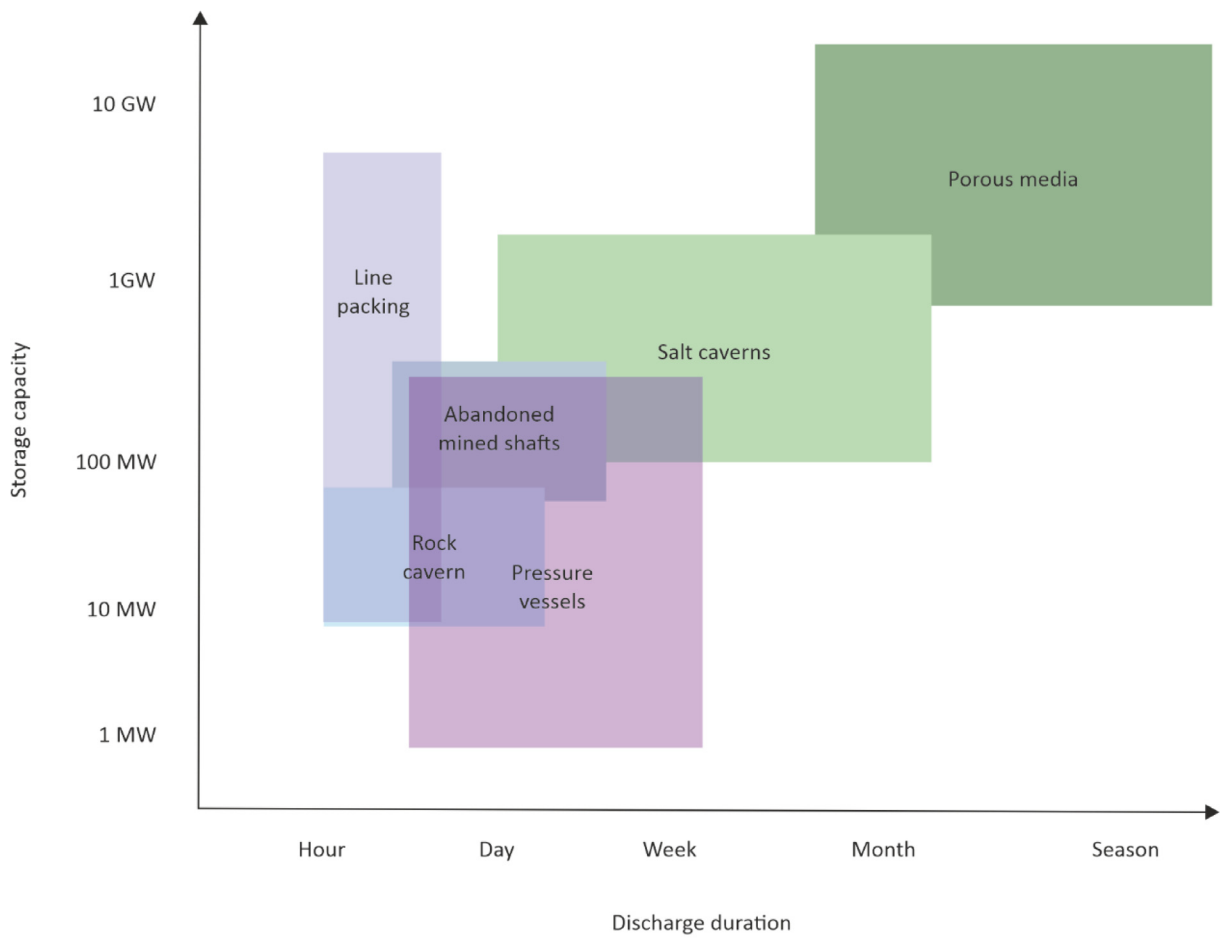
Comparison of storage capacity and discharge duration of above ground and underground technologies.

The underground hydrogen storage (UHS) option is ideal for large-scale storage independent of seasonal fluctuation ( Figure 2) and geographical constraints ^12, 13^ and directly supports the need for decarbonisation in transport, power, heating, and industries. Furthermore, UHS technologies are advantageous compared to surface storage facilities due to 15: 1. Limited footprint, 2. High protection over external influences, 3. High safety due to caverns tightness and a considerable distance from the biosphere and hydrosphere, 4. Large geometrical volumes, high operating pressures, and high storage capacity, 5. Low specific costs and low capital costs to large-scale storage, 6. High availability of natural reservoirs, and 7. Ability to maintain very high operating pressures due to overlying rock thickness.

The available UHS technologies are separated into two major categories; a. the rock caverns, and b. the porous media. The rock caverns are subdivided into 1. salt caverns, 2. engineered rock cavities (lined, unlined, and refrigerated rock caverns), and 3. abandoned mines. The porous media subdivided into 1. depleted hydrocarbon reservoirs, and 2. aquifers and traps. Depending on the category, the technical requirements for the construction of the storage reservoir vary and, by this, determine the cost ^16^. Moreover, TRL for all these methods varies with a giving estimation in Table 2 and as such the resolution analysis offered is quite crude but still offers a basement of discussion.

**Table 2.** Techno-economic feasibility and maturity of each underground hydrogen storage option.

Storage Options	Reservoirs	Maturity	TRL
Host Rocks	Engineered Cavities	Prospective technology, pre-commercial pilots and conceptual designs	6
	Abandoned Mines	Difficult to implement	2
Salt	Salt caverns	Proven technology, sparsely implemented	9
Porous media	Aquifers and Traps	Prospective technology, pre-commercial pilots and conceptual designs	4
	Depleted Hydrocarbon Reservoirs	Prospective technology, pre-commercial pilots and conceptual designs	7

## Technical requirements

The technical requirements differ based on the UHS option. As mentioned above, the two main categories are the rock caverns and the porous media. However, some subcategories of them have common specific needs, and for this study, they are explained together. Thus, the main technologies are divided as follows, and the reasoning of their categorisation for the purpose of this review is given below:

1. Engineered rock cavities, including lined and unlined rock caverns requiring similar excavation procedures.
2. Refrigerated rock caverns require similar procedures as engineered rock caverns. The only difference is the nature of stored gas, which entails specific extra equipment.
3. Salt caverns require different excavation processes.
4. Abandoned mines will be described together with depleted hydrocarbon reservoirs despite belonging to different major categories. In both cases, their previous utilisation has already developed the main infrastructure.
5. Aquifers require a completely different approach from all the other technologies.

Aboveground facilities doesn’t differentiate the cost of the available underground technologies as they are common for all of them and necessary for injecting and withdrawing hydrogen between the surface and the subsurface facilities. The main parts of the aboveground installation include ^17, 18^:

a. Compressor station, required for pressure control,
b. Heating/cooling equipment, aiming to protect from exceeding the maximum operating temperature of the construction while enabling more hydrogen storage,
c. Piping,
d. Valves, regulating the needed flow and balancing the pressure,
e. Metering, and
f. Control system.

A system of tunnels is developed for access to the main cavern when it is necessary ^19^.

### Lined and unlined rock caverns

The excavation of lined, unlined, and refrigerated rock cavities is performed by conventional mining techniques of cutting or blasting and shaft sinking ^20^, requiring the same parameters as in Table 3. The unlined rock caverns refer to the mechanically excavated hard rock caverns, where the roof and the caverns' walls are unlined and covered by fibre-reinforced shotcrete. Unlined caverns allow for minimising construction costs and supply water flows from the surrounding rock ^20^. High pressurised gases need to be stored in constructed caverns with large overburden to balance gas and in-situ rock stress. Gas-tightness balance requires a higher groundwater pressure than the gas pressure in the cavern periphery. If these circumstances are not fulfilled naturally, additional techniques, such as water curtain systems, are used to prevent gas leakage.

**Table 3.** Criteria and requirements for all types of rock caverns ^i^.

Criteria	Requirements
Geology Rock Type	Intrusive igneous rocks, massive sedimentary rocks, massive non-foliated metamorphic rocks
Structure	Homogeneous, isotropic rocks with no discontinuities or significant tectonic deformations
Depth	70 – 200 m or depths where groundwater hydrostatic pressure only slightly exceeds the hydrogen pressure
Porosity	Low < 10 % and preferably < 4 %
Permeability	Low < 100 mD and preferably < 10 mD
Hydraulic Conductivity	< 10 ^-8^ m/sec for water
Thermal Stability	277–353 °K
Storage caverns lifetime	30 years or longer

^i^This table has been reproduced with CCC permission from 10, Elsevier.

**Figure 3.**
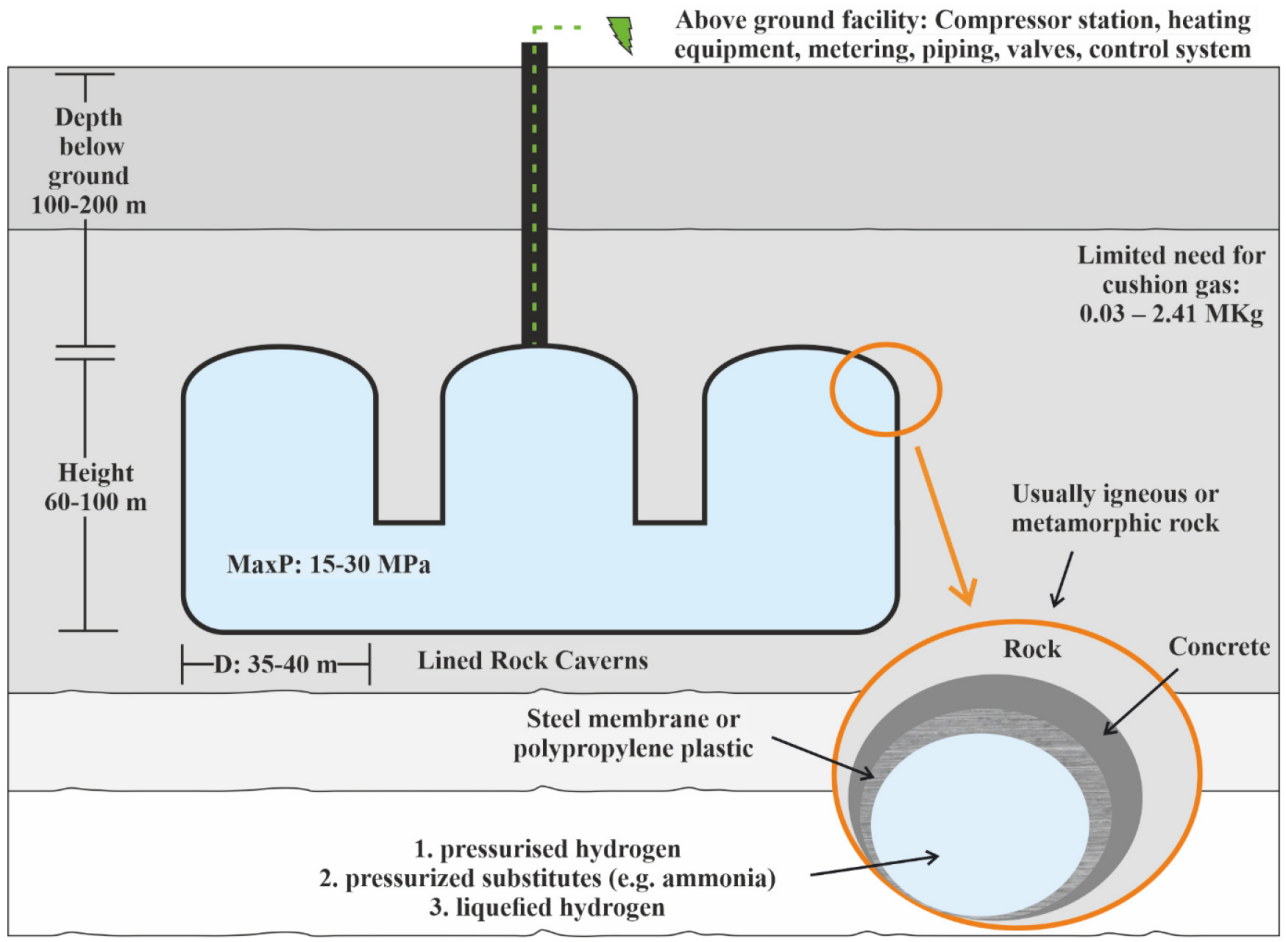
Requirements of Lined Rock Caverns.

The lined rock caverns (LRC) include a steel tank surrounded by concrete to store high-pressurised gas ^17^. The only restriction for selecting the proper geological environment for LRC is the weight of the rock mass to prevent overburden uplifting ^18^. LRC can be composed of one or more caverns with a vertical cylinder shape and rounded tops and bottoms ( Figure 3).

The lining of rock cavern is crucial for gas storage and consists of three main structural components:

a. A sealing layer, able to contain the gas in the construction,
b. A pressure-distributing part to transfer the load created from the gas to the rock mass,
c. A stable rock mass able to carry the created gas pressure.

During the cavern's depressurisation, a groundwater drainage system is needed to decrease the hydrostatic pressure placed around the cavern's perimeter ^19^. The host rock needs to withstand and absorb the created pressure load to avoid the contribution of the lining strain. The concrete layer is designed to transfer pressure from the constructed cavern to the surrounding environment with parallel provisions of a smooth surface for lining placement.

The cavern wall construction may include installing rock bolts or grouting the rock mass. When necessary, the shotcrete can be used to smooth the cavern surface before the lining installation and merge the lining components as slabs and membranes, aiming for a gas-tight seal ^8^. The layered cavern wall can ensure tightness criteria and stability; thus, the actual sealing layer, usually constructed by steel or polymer membrane, does not require very thick. Another solution is developing a steel structure made from reinforcing struts and steel plates by installing cement layers in the annulus between the created structure and the rock mass. The needed steel plates, in this case, must be larger. In both cases, groundwater must be kept from storage to avoid buoyancy forces and corrosion ^8^.

To avoid leakage, permeability and groundwater control methods are used. Full-scale permeability control techniques are not yet established, but steel-line or frozen storages are potential alternatives. Groundwater control can be achieved by natural groundwater pressure or by artificial methods such as a water curtain system ^21^.

The water curtain system, potentially required in both lined and unlined rock caverns, aims at ^22^:

a. Maintenance of water saturation of the rocks fracture system under the excavation process.
b. Water pressure regulation to avoid gas breakthrough.
c. Gas storage at high feasible pressures than typically exists due to artificially high-water pressure surrounding the cavern.

The main design of water curtain techniques is water flowing from outside toward the cavern goal, avoiding escape and migration of the stored gas from the cavern ^21^. The technique includes a series of horizontal boreholes drilled above the rock chamber surrounding the excavated cavern. The holes allow the operation of water pressure higher than the air pressure in the cavern, protecting the gas leakage via the surrounding rock mass. A typical water curtain system consisted of 21:

i. Boreholes' spacing, between 5 to 20 m
ii. Boreholes' distance from the storage cavern.
iii. Water curtain extent.
iv. The water pressure curtain (or potential) correlated to the storage pressure (potential).

Although the water curtain design is conceptually simple, it can be a demanding technical task due to the nature of the host rock and its special traits. In addition, when the water curtain technique is required, the installation cost increases when difficulties occur due to the irregular nature of rock fractures.

### Refrigerated rock caverns

**Figure 4.**
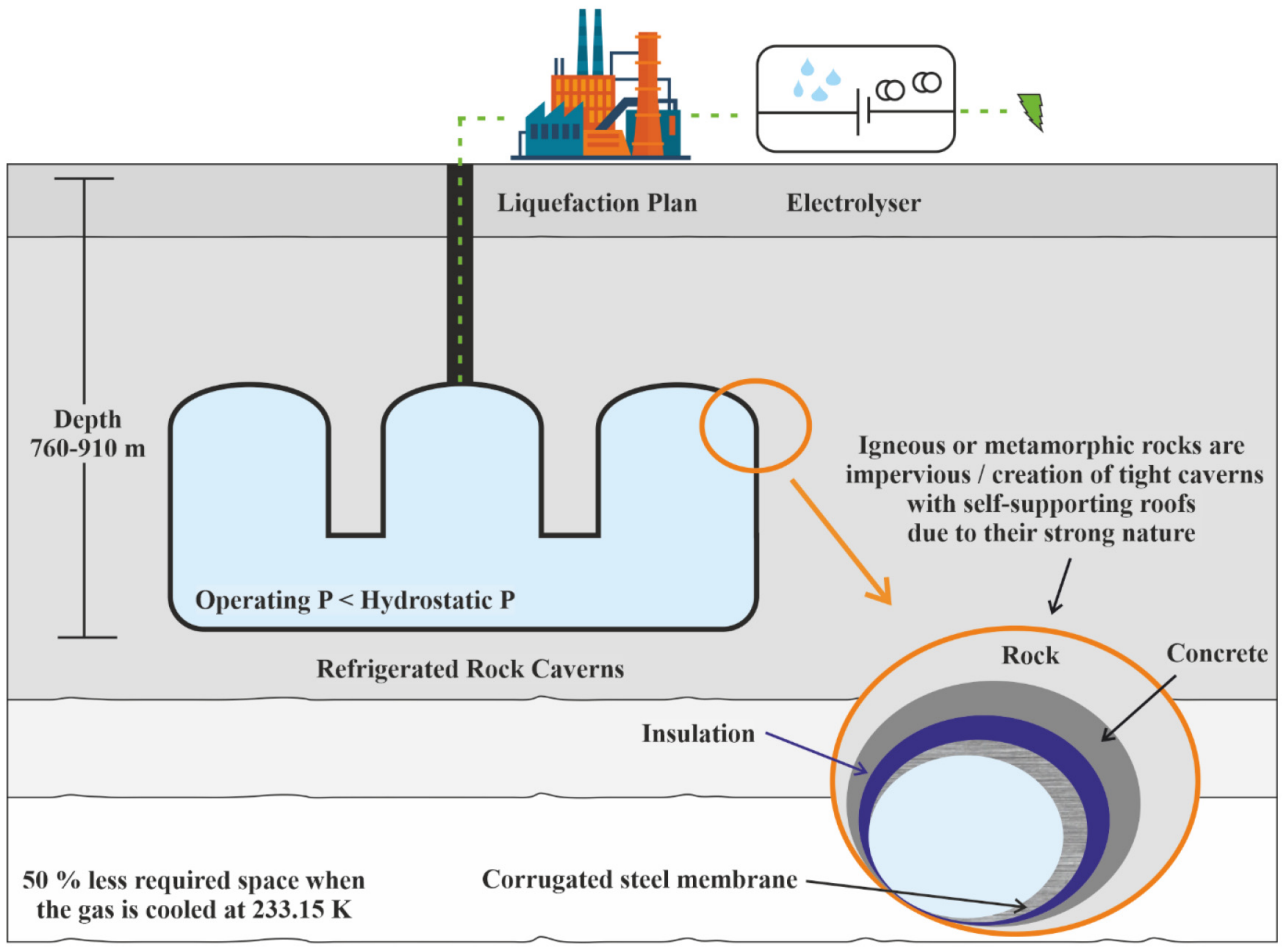
Requirements of Refrigerated Rock Caverns.

The refrigerated cavern method aims at the massive decrease in the specific volume of the gas due to the cooling down of the storage medium prior to its emplacement to the cavern ( Figure 4). Their construction was performed by the same methods described in lined and unlined rock caverns. Due to the different nature of stored gas, slight differences exist that correspond to different costs. The refrigerated caverns are more advantageous than the other engineered rock cavities because the required volume is significantly lower. Chilling of H _2_ and its compression reduces the required storage space ^24^.

Despite the relatively lower construction cost, the storage medium's hydrostatic pressure must be greater than the storage pressure due to its partial evaporation ^8^. The need for the extremely low temperature of liquid hydrogen requires specific infrastructure for achieving such temperatures and is extremely energy-intensive, which may be uneconomical. Cooling the cavern below the groundwater's freezing point is a possible way to strengthen the sealing effect. Such a technique involves the installation of cooling pipes within the rock mass; thus, the cost is also increasing ^8^.

### Salt caverns

Salt caverns are artificially constructed chambers on salt formations opened by solution mining, also known as leaching ^25^. Fresh or low-salinity water is injected into the salt bedrock via an established borehole to dissolve the salt. Usually, a volume of 7 to 8 m ^3^ of fresh water is needed for the salt dissolution of 1 m ^3^ ^26^. As the opening of salt caverns is slightly differentiating by other rock caverns, the mandatory criteria for solution-mined salt caverns are given in Table 4.

**Table 4.** Criteria and requirements for salt caverns ^i^.

Criteria	Requirements
Geology Rock Type	Salt formations
Structure	Salt domes or Bedded salt with 95 % halite
Caprock	Anhydrite, gypsum, limestone
Depth	200–2000 m
Height (H)	Around 300 m
Diameter (D)	Around 70 m
Height/Diameter Ratio (H/D)	5–20 and typically 5
Distance between caverns (S)	Around 300 m
Separation/Diameter Ratio (S/D)	2–10 and typically > 4
Maximum salt temperature	80 °C
Operating Pressure	24 to 80 % of the overburden pressure
Storage volume	300000–750000 m3 for salt domes and 100000 m3 for bedded salt
Salt caverns lifetime	30–50 years

^i^ This table has been reproduced with CCC permission from 10, Elsevier.

Cavern development influenced by:

a. Water injection rate, and
b. The water injection and brine removal equipment location.

The leaching process is divided into four main stages ^27^:

a. Sump leaching, where the necessary space is developed in the deepest part of the chamber.
b. Leaching of the main chamber, where the target shape and storage capacity are determined.
c. Leaching of the cavern dome ensures the geomechanical stability of the construction, and
d. Neck leaching, including the dome connection with the cemented column casing pipes.

The next step involves the debrinning process by injecting hydrogen into the cavern. An outer pipe is used for the gas injection, while an inner leaching pipe extracts the brine. Then, both leaching pipes are pulled out of the hole. A full characterisation of brine is proposed before its disposal to the environment. Three main options are proposed for its safe management:

a. Pumping to the sea,
b. Disposal into salt aquifers, and
c. Use as a raw material for salt production in the chemical industry.

The distance of salt caverns by sites available for brine disposal is crucial as it can significantly increase the cost.

**Figure 5.**
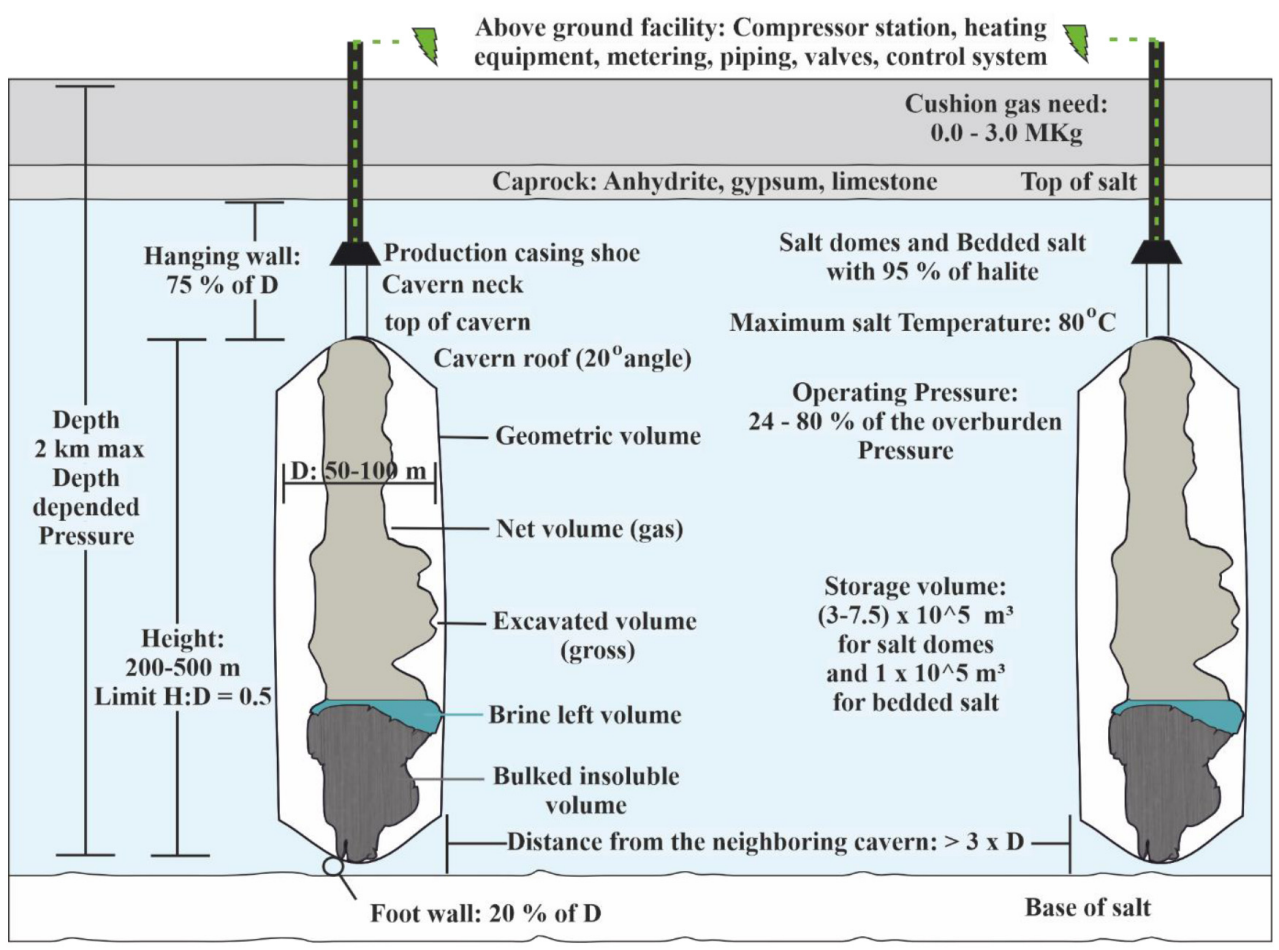
Salt caverns requirements, where H is height and D is diameter.

The developed volume and the operating pressure affect the cavern's capacity, while the cavern's depth influences the operating pressure. The main requirements for salt caverns are given in Figure 5 ^10^. A well combined with the salt cavern can fill the cavity with gas and allow the performance of gas tightness studies and operational procedures tests that will ensure the safety of the caverns.

When the leaching process is used, three main conditions are required:

a. The lack of high content of insoluble substances characterises salt formation with efficient structure and thickness in a proper depth.
b. Efficient fresh water supply for solution mining.
c. An environmentally friendly way for brine management and disposal, ensuring low-cost maintenance.

### Abandoned mines and depleted hydrocarbon reservoirs

In the case of abandoned mines and depleted hydrocarbon reservoirs, the initial construction aimed to extract natural resources, not gas storage. Thus, the available storage volume is fixed and only slight changes can occur. Moreover, the stages of geological investigation and exploitation are missing in these options, which minimises the cost. Despite the lack of cavern excavation, specific requirements must be fulfilled to ensure the stability of gas storage.

More specifically, abandoned mines require a homogeneous rock type with tight and stable characteristics ^28^ to construct a sealing structure in the existing shafts. Where necessary, the water curtain technique could be used. The excavation and tunnelling of the mine should have caused limited damage to the remaining rock mass and preferably performed by room and pillar excavation or circular room and pillar excavation, as longwall mining or other techniques can cause fractures to the rock formation. For the drilling method, drilling and blasting are not preferable and milling, scraping, or drilling tools could be used. The smaller the number of access drifts and shafts, the preferred. A high-water table is needed if the storage space is to be sealed by groundwater management ^8^. The stored gas may interact with the remaining minerals in the abandoned mines, resulting in failures ^9^. Depleted coal mines can reduce the volume of potentially stored gas due to adsorption ^29, 30^.

**Figure 6.**
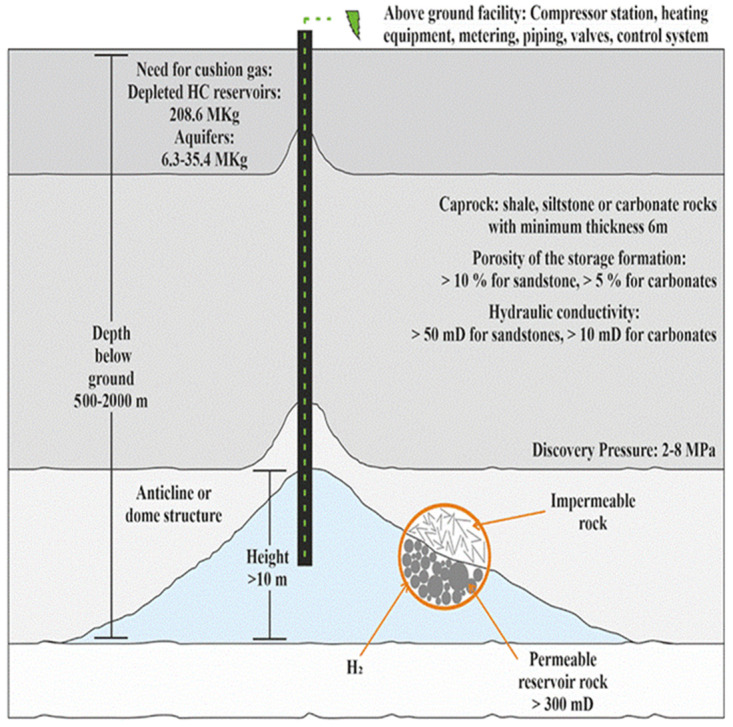
Requirements of porous media.

For depleted hydrocarbon reservoirs, pre-existing infrastructures could be used after appropriate modifications ^31, 32^. Also, in this case, the hydrogen mixing with the remaining hydrocarbons must be investigated to avoid the loss of hydrogen purity ^31^. In some cases, depending on the nature of the remaining hydrocarbons, they could be used as cushion gas, providing a lower cost ^33^. The remaining water in the reservoir, a residue of previous processes, must be removed before the implementation ^34^. The technical characteristics of the boreholes, especially the steel, cement materials, and type of casing, are crucial for the safe implementation of UHS in depleted hydrocarbon reservoirs ^33^. Trunks and pipelines of the surface installations are sometimes suitable for storing monitoring systems and thus decrease the cost ^35^. Specific requirements for depleted hydrocarbon (HC) reservoirs are given in Figure 6.

### Aquifers

Saline aquifers are porous sedimentary rock with specific parameters, the same for depleted hydrocarbon reservoirs, as given in Figure 6 ^10^ and summarised in Table 5. They are saturated in saline water and can potentially be used for injecting hydrogen ^36^.

**Table 5.** Criteria and requirements for porous media (aquifers and depleted hydrocarbon fields) ^i^.

Criteria	Requirements
Geology Rock Type	Sandstones and conglomerates
Caprock	Shale, siltstone and carbonate rocks with a minimum thickness 6 m
Structure	Anticline or dome
Depth	200-2000 m
Porosity	≥ 10 %
Permeability	> 300 md
Vertical Closure	≥ 10 m
Thickness	≥ 10 m
Discovery Pressure	2-8 MPa
Porous media lifetime	> 50 years (needs proof of concept)

^i^This table has been reproduced with CCC permission from 10, Elsevier with additions.

During the injection period, the water is displaced by the available pores, which gives free space for hydrogen storage. The gas injection is performed under increasing pressure conditions, and the brine refills the empty pores during the gas withdrawal ^33^. The volume of the stored hydrogen can reach up to hundreds of Mm ^3^ and is strongly connected with the porous media's temperature, pressure, volume, and porosity ^37^. The gas dissolution in saline aquifers must be thoroughly tested to avoid gas losses. Gas dissolution is affected by P-T conditions and is possible in the contact area of hydrogen in saline water systems. Increasing hydrogen solubilities may also result from circulation processes in the brine-gas interface that replace the hydrogen/saturated brine with unsaturated brine ^38^. An extensive geological survey must take place before the injection to avoid the migration and leakage of hydrogen by faults and fractures or voids existing in the neighbouring environment or produced by mineralogical interactions ^33^. The geological survey increases slightly the cost.

## Economic parameters

The basic economic parameters for the development and operation of UHS options are extensively examined in this section. The given costs are transferred to on-date values based on inflation rates for the dollar that is based on the latest US government Consumer Price Index data, based on the following equation:

$$Inflation\;Rate\;=\;\frac{B\;–\;A}{A}\;\times \;100$$

where A is the starting Cost, and B is the ending cost. The formula requires the starting point of a specific year in the past in the consumer price index for a specific good or service correlated with the current recording for the same good or service in the consumer price index. Euros did not require such transformations as their difference with on-date values is negligible (< 50 €). Euros converted to dollars for comparable reasons. For the economic assumptions, sensitive parameters to fluctuations in market conditions, such as changes in the price of hydrogen or the cost of electricity, did not used.

## Cushion gas needs

Cushion gas, also known as base gas or dead gas ^31^, is an important parameter for maintaining the appropriate pressure, stability, and water intrusion within the reservoir ^15, 35, 39^. Cushion gas cannot be used for energy purposes, and the needed amount depends on the technology from 25 to 75 % ^39^. The cushion gas is related to the pre-injection of the hydrogen into the geological formation prior to the implementation of UHS. It is inactive, non-recoverable and specifies the volume and pressure conditions of the reservoir ^40^. The most widely used gasses are CO _2_, N _2_, and CH _4_.

The lowest cushion gas volume is required for salt caverns and depleted hydrocarbon reservoirs, while the highest is required for the aquifers, as shown in Table 6. Remaining hydrocarbons in the case of depleted hydrocarbon reservoirs can be used as cushion gas, depending on their nature ^41^. Thus, the cost required for cushion gas decreases.

**Table 6.** Cushion gas requirements for every UHS technology.

Hydrogen (H _2_) Storage Capacities	Salt Caverns	Aquifers	Depleted Gas Fields	Rock Caverns	Abandoned Mines
Cushion gas [MKg]	0.0 – 3.0	6.3 – 35.4	208.6	0.0 – 2.4	0.1 – 46.8
Cushion gas [Mm ^3^ (st)]	0.0 – 34.9	73.6 – 415.3	2.5	0.4 – 28.3	1.58 – 550
Cushion gas [GWh]	0.0 – 117.2	247.0 – 1393.1	8215	1.3 – 94.8	5.29 – 1844
Costs for cushion gas [M$]	0.0 –7.3	15.3 – 86.3	508.7	0.1 – 5.9	0.3 – 114.2

### Unlined rock caverns

The estimated cost for engineered rock cavities is strongly connected with the quality of the rock. Therefore, the needed equipment with the derived cost is given in Table 7 ^8, 22^ for good rock conditions. Moreover, the estimation of cushion gas cost and capital cost for the unlined rock cavern of Haje, Czech Republic, is included.

**Table 7.** Unit prices and cost analysis of hydrogen storage in unlined rock caverns ^ii^.

Unit	Description	US
Access tunnel	45 m ^2^, 1:8	1930.0 $/m
Ventilation shafts	20 m ^2^, vertical	5790.0 $/m
Water tunnels	15 m ^2^	1158.0 $/m
Water curtain drillholes	Percussion	30.0 $/m
Top galleries	160 m ^2^, 20 bolts m ^-1^ shotcrete	5790.0 $/m
Benches	11 m (height)	17371.0 $/m ^3^
Rock haulage	Trucks to adit	0.8 $/ton km
Large holes	Ø200 mm, cased	772.0 $/m
Installations in rock	Compressor station excluded	2.3 – 3.0 M$
Reinforcement, grouting, plugs, surface works, extras		15.4 – 19.3 M$
Haje (Czech Republic)		
Geology	Granite	
Operator	RWE Transgas	
Seal/Lining	In-situ	
Reference depth	950 m	
Geometrical volume/CapEx	620000 m ^3^	160.0 $/m ^3^
Maximum/Minimum pressure	12.5/3.8 MPa	
Cushion gas/Cost	69.7 GWh ^1^	4.3 M$ ^1, 2^ / 26.4 $/kg
Working gas/ CapEx	148.7 GWh ^1^	2.2 $/m ^3^(st) /634.8 $/GWh ^1^
Injection-Withdrawal/ CapEX for storage	22500-33700 kg/h	99.7 M$

^1^ Energy related to upper heating value (14.72 kWh/kg for natural gas, 39.39 kWh/kg for hydrogen); ^2^ Energy costs of 27.0 $/MWh for natural gas and 43.3 $/MWh for hydrogen electrolysis, EEX/GASPOOL EEX/ELIX 200 d average prices ^ii^ This table has been reproduced with CCC permission from 22, Elsevier.

### Lined rock caverns

Aboveground facilities for LRC are similar to a pipe storage system. For the estimation of the required cost, it was supposed that the facility is located one mile from the storage site. Compressors, valves, and pipelines that were constructed with right-of-way related costs were estimated, as given in Table 8 ^42, 43^.

**Table 8.** Aboveground capital, operational and maintenance costs for Lined Rock Caverns with a storage capacity of 500 tons for a labour cost of 33.5 h and 5.74 cents/k Wh electricity ^iii^.

Description	Value
Storage capacity (kg)	5x10 ^5^
Cost of hydrogen cushion gas ($/kg)	5.0
Need of cushion gas (kg)	8.4 x10 ^4^
Installed compressor cost (M$)	3.5
Pipe to H _2_ production facility (M$)	0.8
Valves and instrumentation (M$)	0.1
Total cushion gas cost (M$)	0.4
Land cost (M$)	0.3
Total aboveground costs (M$)	5.1
Labor cost (M$/year)	0.3
Maintenance cost (M$/year)	0.2
Electricity cost (M$/year)	<0.1
Property tax and insurance (M$/year)	0.1
Total operational and maintenance costs (M$/year)	0.6

^iii^ This table has been reproduced with CCC permission from 43, Elsevier.

The storage of 500 t hydrogen in LRC with a constant minimum pressure of 2 MPa and a range of maximum storage pressure between 7.5 and 30 MPa is given in Table 9. Almost 66 % of the capital cost corresponds to an underground facility, 14 % to aboveground facilities (including the equipment given in Table 8), and 21 % to miscellaneous costs. For this type of technology, the underground facilities represent the higher cost. When the pressure increases, the capital cost decreases at a slower rate, between 15–20 MPa. A 21 % cost was saved for a 30 MPa storage installation compared to 15 MPa.

**Table 9.** Breakdown of capital costs for underground Lined Rock Caverns ^iv^.

Maximum Storage Pressure (MPa)	7.5	10	15	20	25	30
Dome water volume (x10 ^4^ m ^3^)	12	8.1	5.1	3.8	3.1	2.6
Concrete volume (x10 ^4^ m ^3^)	2.6	2.1	1.5	1.3	1.1	1.0
Shaft ($/kg)	11.2	11.2	11.2	11.2	11.2	11.2
Dome ($/kg)	54.0	38.4	25.0	19.0	15.6	13.4
Liner ($/kg)	9.1	7.2	5.4	4.6	3.9	3.6
Concrete ($/kg)	8.5	6.7	5.0	4.2	3.6	3.3
Total underground costs ($/kg)	82.8	63.5	46.6	38.9	34.3	31.4
Engineering ($/kg)	12.4	9.5	7.0	5.8	5.1	4.7
Contingency ($/kg)	8.3	6.4	4.7	3.9	3.4	3.1
Permitting ($/kg)	2.5	1.9	1.4	1.2	1.0	0.9
Geological survey ($/kg)	2.3	2.3	2.3	2.3	2.3	2.3
Total other costs ($/kg)	25.5	20.1	15.4	13.2	11.9	11.1
Compressor ($/kg)	5.0	5.8	7.1	7.8	8.5	9.2
Piping, instrumentation, valves ($/kg)	0.9	1.2	1.7	2.2	2.7	3.1
Cushion gas ($/kg)	1.0	0.7	0.4	0.3	0.3	0.2
Land cost ($/kg)	0.7	0.7	0.6	0.6	0.6	0.6
Total aboveground costs ($/kg)	7.6	8.3	9.8	11.0	12.1	13.1
Total cost ($/kg)	115.8	91.9	71.8	63.1	58.3	55.5

^iv^This table has been reproduced with CCC permission from 43 Elsevier.

In another estimation, the capital cost and the unit cost involved the total erected capital costs, operating expenses and plant cost, as shown in Table 10.

**Table 10.** Installed and unit cost for Lined Rock Caverns ^v^.

Basic parameters		
Injection rate	159.7 tonnes/day	
Power $/MWh	59.8	
Service factor	0.36	
Annual Return of Investment (ROI) %	15	
Description	Cost (M$)	
Cost of cavern development and well-head equipment	32.9	
Three-stage reciprocating compressor, c/w 6000 HP electric drive (Total for seven operating units and one spare)	55.1	
Foundations and erection	5.4	
20 MW transformer and primary breaker (Total for two units, installed)	8.1	
Building, c/w heating, ventilation, lighting and gas monitoring and alarm system	4.2	
Gas holder, 14000 Nm ^3^ capacity	2.1	
Interconnecting pipework	2.7	
Total installed cost	110.5	
Operating expenses	Unit cost ($)	Annual cost (M$)
Electrolytic hydrogen, 159.71 tonnes/day	6143.1	358.1
Power, 28.00 MWh/h	59.8	5.2
Cooling water, 7474 tonnes/day	0.2	0.2
Utilities		0.8
Operating personnel (shift), 16 @ 77794.3 $/y		1.3
Supervision and fringe benefits @ 55 % of the labour		0.7
Administration @ 20 % of labour and supervision		0.4
Maintenance materials and labour @ 2 % of capital cost		2.2
Other @ 2.5 % of capital cost		2.8
Total operating expenses		371.7
Investment		Annual cost (M$)
Total erected capital cost		110.4
Land cost, 2 ha		0.2
Engineering costs, 10 % of erected cost		11.0
Total plant cost		121.6
Cushion gas, 425.71 tonnes @ 6143.1 $/tonne		2.6
Working capital, 30 days of operating expenses		30.5
Start-up cost, 2 % of total plant cost		2.4
Interest		
before start-up @ 12% per year		
Year 1 40 % of total plant cost, 12.4 M$		
Year 2 60 % of total plant cost, 8.8 M$		21.1
Total investment		178.2

^v^This table reproduced with CCC permission from 40, Elsevier, modified with inflation rate.

### Refrigerated rock caverns

An estimation of the central installation for a refrigerated mined cavern with a delivery pressure of 2.41 MPa during the injection is given in Table 11 ^24^. The annual operating costs for such a facility are also given in Table 11, while other costs differentiating by the site are the taxes, insurance, depreciation, management, working capital as well as other overhead charges ^24^.

**Table 11.** Cost summary of refrigerated rock mined cavern ^vi^.

Details	
Working Gas Storage (Billion Standard Cubic Feet)	1.524
Maximum Injection Rate (MMSCFD)	250
Maximum Withdrawal Rate (MMSCFD)	250
Injection Cycle (days)	20
Withdrawal Cycle (days)	20
Deep mine (m)	914.4
Description	Cost (M$)
Conventional mining and shaft sinking (6292335 bbls of mining mobilisation)	205.4
Refrigeration system, compressors, and process	58.5
Electrical and instrumentation equipment	4.1
Compressor building (64.08 m x 15.24 m), foundation, slab, and control	1.8
Control building foundation, concrete supports for compressor station piping, mechanical separators foundation, engineered aluminum oxide pellet, and pressure-reducing station foundations	0.4
Gas storage facility final design (8 months duration)	2.0
Gas storage facility construction and commissioning	8.8
Subtotal	281.0
Contingency on All costs 10%	28.1
Subtotal	309.1
Contractors' Profit at 10 % on Surface Facilities	6.5
Total Project cost	315.6
Cost of facility per MMSCF of base gas storage ($)	63.1
Mining cost per barrel of mined space ($)	36.6
Cost Parameter	Annual cost ($)
**Labor**	
1 Supervisor	179685
8 Shift Operators	893685
2 Relief Operators	201080
1 Clerical	68223
**Labor Subtotal**	**1342673**
Maintenance Material & Labor	219395
Operating Supplies	65819
Energy (as fuel gas assumes $ 4.57 per MMBtu)	2000918
Total	3628805

^vi^ This table has been reproduced with permission for any use from 24, PB-KBB INC.

The capital cost and unit cost derived from operating expenses, total erected capital cost, and plant cost for the storage of liquified hydrogen are given in Table 12 ^40^.

**Table 12.** Installed and unit cost for refrigerated mined caverns ^vii^.

Basic parameters		
Delivery rate	29.5 tonnes/day power $/MWh	
Power $/MWh	59.8	
Service factor	0.9726 (355 days/y)	
Annual capital charge	15 % (incl. depreciation, interest and profit)	
Description	Cost (M$)	
40 t/day (36 × 10 ^3^ kg/day) hydrogen liquefaction unit	96.6	
Double-walled, vacuum-perlite insulated spherical tanks, each capable of storing – 300000 kg liquid hydrogen (LH _2_)	40.4	
Cold hydrogen gas storage tank - 1250 Nm ^3^	4.8	
Hydrogen gas buffer tank - 14000 Nm ^3^	2.01	
LH ^2^ Fuelling system, including pumps, piping, valves	68.8	
Interconnecting piping, valves for the storage tanks	6.0	
Foundations and installation for the tanks	6.0	
Sub-total	224.6	
Contingency allowance (10 %)	22.5	
Total installed Cost	247.1	
Operating expenses	Unit cost ($)	Annual Cost (M$)
Electrolytic hydrogen, 30.31 tonnes/day	4040.2	43.5
Power, 25.00 MWh/h	59.8/ MWh	12.8
Utilities (chemicals, nitrogen, water, etc.)		4.4
Operating personnel, 9 @ 77794.3 $/y		0.7
Supervision and fringe benefits @ 55 % of labour		0.4
Administration @ 20 % of labour and supervision		0.2
Maintenance materials and labour @ 2 % of capital cost		4.9
Other @ 2.5 % of capital cost		6.2
Total operating expenses		73.1
Investment		Annual Cost (M$)
Total erected capital cost		247.2
Land cost, 4 ha		0.3
Engineering costs, 6 % of erected Cost		14.8
Total plant cost		262.3
Non-recoverable liquid, 100 tonnes @ 4040.19 $/tonne		0.4
Working capital, 30 days of operating expenses		6.0
Start-up cost, 3 % of total plant cost		7.9
Interest		
before start-up @ 12 % per year		
Year 2 40 % of total plant cost, 12.6 M$		52.6
Total investment		329.2

^vii^ This table reproduced with CCC permission from 40, Elsevier, modified with inflation rate.

### Salt caverns

A cost estimation was performed for storing 500 t hydrogen in salt caverns for a range of depths between 450 and 1200 m. A minimum content of cushion gas was used to estimate capital cost. Thus, a percentage of 30 % cushion gas was used for calculations. Almost 49 % of the capital cost corresponds to activities related to underground facilities, 24.5 % to aboveground facilities and the remaining 26.5 % to costs derived by brine-related activities, as shown in Table 13. Costs related to brine disposal are affected by local political conditions and environmental laws, resulting in site differentiation ^43^.

**Table 13.** Breakdown of capital costs for hydrogen storage in salt caverns ^viii^.

Cavern Roof Depth (m)	457	609	762	914	1067	1219
Maximum storage pressure (MPa)	7	10	12	14	17	19
Minimum storage pressure (MPa)	2.2	2.9	3.5	4.1	4.7	5.3
Cavern water volume (m ^3^)	125897	96185	78294	67634	59287	52973
Cushion gas (%)	31.1	31.1	30.7	30.8	30.8	31.0
Drill + casing ($/kg)	3.3	4.4	5.6	6.7	7.8	8.9
Leaching ($/kg)	5.1	4.5	4.1	4.0	3.8	3.8
Mechanical integrity test ($/kg)	2.3	2.3	2.3	2.3	2.3	2.3
Total construction costs	10.7	11.2	11.9	12.9	13.9	15.0
Geological survey ($/kg)	2.3	2.3	2.3	2.3	2.3	2.3
Engineering ($/kg)	1.6	1.7	1.8	1.9	2.1	2.2
Contingency ($/kg)	1.1	1.1	1.2	1.3	1.4	1.5
Permitting ($/kg)	0.3	0.3	0.4	0.4	0.4	0.4
Total engineering costs ($/kg)	5.3	5.4	5.6	5.9	6.2	6.5
Brine transportation ($/kg)	7.0	5.3	4.3	3.7	3.3	2.9
Brine disposal ($/kg)	7.0	5.3	4.3	3.7	3.3	2.9
Total brine disposal costs ($/kg)	13.9	10.6	8.7	7.5	6.6	5.9
Compressor ($/kg)	5.0	5.7	6.3	6.9	7.4	7.8
Piping and instrumentation ($/kg)	1.0	1.0	1.0	1.0	1.0	1.0
Cushion gas ($/kg)	1.4	1.4	1.3	1.3	1.3	1.3
Land cost ($/kg)	0.8	0.7	0.7	0.7	0.7	0.6
Total aboveground costs ($/kg)	8.1	8.8	9.4	9.9	10.4	10.8
Total cost ($/kg)	38.0	36.0	35.6	36.2	37.0	38.1

^viii^ This table has been reproduced with CCC permission from 43.

The total installed cost estimated involving the cavern and well development, the compressor, pipelines and other required systems, as well as the unit cost derived by operating expenses, total erected capital cost, and plant cost, are given in Table 14 ^40^.

**Table 14.** Installed and unit cost for salt caverns ^ix^.

Basic parameters		
Injection rate (tonnes/day)	159.71	
Power $/MWh	59.8	
Service factor (two tidal periods of 4.5 h/25 h)	0.36	
Annual ROI %	15	
Description	Cost (M$)	
Cost of cavern development and well-head equipment	16.5	
Three-stage reciprocating compressor, c/w 6000 HP electric drive (Total for seven operating units and one spare)	55.1	
Foundations and erection	5.5	
20 MW transformer and primary breaker (Total for 2 units, installed)	8.0	
Building, c/w heating, ventilation, lighting and gas monitoring and alarm system	4.1	
Gas holder, 14000 Nm ^3^ capacity	2.1	
Interconnecting pipework	2.7	
Total installed Cost	94.0	
Operating expenses	Unit cost ($)	Annual Cost (M$)
Electrolytic hydrogen, 159.71 tonnes/day	6143.1	358.1
Power, 28.00 MWh/h	59.8	5.2
Cooling water, 7474 tonnes/day	0.2	0.2
Utilities		0.8
Operating personnel (shift), 16 @ 77794.3 $/y		1.3
Supervision and fringe benefits @ 55 % of labour		0.7
Administration @ 20 % of labour and supervision		0.4
Maintenance materials and labour @ 2 % of capital cost		1.9
Other @ 2.5 % of capital cost		2.3
Total operating expenses		370.9
Investment		Annual Cost (?$)
Total erected capital cost		93.8
Land cost, 2 ha		0.2
Engineering costs, 10 % of erected Cost		9.4
Total plant cost		103.4
Cushion gas, 386.60 tonnes @ 6143.1 $/tonne		2.4
Working capital, 30 days of operating expenses		30.5
Start-up cost, 2 % of total plant cost		2.1
Interest		
before start-up @ 12 % per year		
Year 1 40 % of total plant cost, 3.5 M$ 10.5 M$		
Year 2 60 % of total plant cost, 7.5 M$		18.0
Total investment		156.4

^ix^ This table reproduced with CCC permission from 40, Elsevier, modified with inflation rate.

### Abandoned mines

As abandoned mines do not require the development of a new cavern, but only the investigation of the sufficient nature of the cavern for the storage of gas, a complete cost estimation for the development of the implementation is missing. Thus, only estimations for the needed cushion gas cost were available in the literature, as given in Table 15 ^8^.

**Table 15.** Cushion gas operating characteristics and cost estimation for known abandoned mines.

Operating characteristics	Bernsdorf (Germany)	Leyden (USA)
Geology	Carnalit	Bituminous Coal
Operator	VNG	-
Seal/Lining	Rock salt	Groundwater
Reference depth (m)	600	225
Geometrical volume (m ^3^)	135000	5100000
Maximum pressure (MPa)	50	17.2
Minimum pressure (MPa)	12.4	6.895
Cushion gas (GWh) ^10^	5.29	114.1
Working gas (GWh) ^10^	15.58	168.7
Injection (kg/h)	3600	3600
Withdrawal (kg/h)	3600	3600
Cost of cushion gas (M$) ^10, 12^	0.3	7.1

**NOTES** ^1^ Energy related to upper heating value (14.72 kWh/kg for natural gas, 39.39 kWh/kg for hydrogen); ^2^ Energy costs of 27.0 $/MWh for natural gas and 43.3 $/MWh for hydrogen electrolysis, EEX/GASPOOL EEX/ELIX 200 d average prices

### Depleted hydrocarbon reservoirs

Cost estimation for the cushion gas required in depleted hydrocarbon reservoirs is given in Table 16 ^8^.

**Table 16.** Cost estimation for the depleted hydrocarbon reservoir of Rheden.

Parameters	Rheden (Germany)
Geology	Dolomite
Operator	ASTORA
Seal/Lining	Cap rock
Reference depth (m)	1900-2100
Geometrical volume (m ^3^)	32797585
Maximum pressure (MPa)	280
Minimum pressure (MPa)	110
Cushion gas (GWh) ^1^	8215
Working gas (GWh) ^1^	12322
Injection (kg/h)	125.800
Withdrawal (kg/h)	215.700
Cost of cushion gas (M$) ^1, 2^	508.3
CapEX for storage (M$)	406.0
CapEX per geometrical volume ($/m ^3^)	12.3
CapEX per working gas ($/kg)	1.3
CapEX per working gas ($/m ^3^(st))	0.1
CapEX per working gas ($/GWh) ^1^	31.2

**NOTES** ^1^ Energy related to upper heating value (14.72 kWh/kg for natural gas, 39.39 kWh/kg for hydrogen); ^2^ Energy costs of 27.0 $/MWh for natural gas and 43.3 $/MWh for hydrogen electrolysis, EEX/GASPOOL EEX/ELIX 200 d average prices

The capital cost estimation involving all the necessary facilities and the unit cost are given in Table 17

**Table 17.** Capital and unit cost of depleted gas well storage ^x^.

Basic parameters		
Injection rate (tonnes/day)	112.76	
Power $/MWh	59.8	
Service factor	0.5	
Annual ROI %	15	
Description	Cost (M$)	
Three-stage reciprocating compressor, c/w 5000 HP electric drive (Total for two operating units)	12.0	
Foundations and erection	1.2	
8 MW transformer and primary breaker, installed	2.1	
Building, c/w heating, ventilation, lighting and gas monitoring, and alarm system	1.2	
Gas holder, 14000 Nm ^3^ capacity	2.1	
Interconnecting pipework to compressors	0.9	
New wells (27 total), complete with well-head equipment and interconnecting pipework	6.0	
Total installed cost	25.5	
Operating expenses	Unit cost ($)	Annual Cost (M$)
Electrolytic hydrogen, 112.76 tonnes/day	4380.4	90.2
Power, 7.00 MWh/h	59.8	1.8
Cooling water, 1900 tonnes/day	0.2	0.1
Utilities		0.8
Labour, 7 @ 77794.3 $/y		0.5
Supervision and fringe benefits @ 55 % of labour		0.3
Administration @ 20 % of labour and supervision		0.2
Maintenance materials and labour @ 2 % of capital cost		0.5
Other @ 2.5 % of capital cost		0.6
Total operating expenses		95.0
Investment		Annual Cost (M$)
Total erected capital cost		25.4
Land cost, 2 ha		0.2
Engineering costs, 10 % of erected cost		2.5
Total plant cost		28.1
Cushion gas, 13217 tonnes @ 4380.4 $/tonne		57.9
Working capital, 182.5 days of operating expenses		47.5
Start-up cost, 2 % of total plant cost		0.6
Interest		
before start-up @ 12 % per year		
Year 2 40 % of total plant cost, 2.0 M$		4.9
Total investment		139.0

^x^ This table reproduced with CCC permission from 40, Elsevier, modified with inflation rate.

### Aquifers

As the technology for aquifers is still in primary steps, only estimations about cushion gas costs are available in the literature, as given in Table 18 ^8^.

**Table 18.** Estimated cushion gas for aquifers.

Parameters	Hähnlein (Germany)	Stenlille (Denmark)
Geology	Sandstone	Sandstone
Operator	E.On	DONG
Seal/Lining	Claystone	Cap rock
Reference depth (m)	500	1500
Geometrical volume (m ^3^)	3198282	4141615
Maximum pressure (MPa)	53	170
Minimum pressure (MPa)	39	150
Cushion gas (GWh) ^1^	247.0	1393.1
Working gas (GWh) ^1^	247.0	750.1
Injection (kg/h)	7200	30700
Withdrawal (kg/h)	9000	18000
Cost of cushion gas (M$) ^1, 2^	15.3	86.2

NOTES ^1^ Energy related to upper heating value (14.72 kWh/kg for natural gas, 39.39 kWh/kg for hydrogen); ^2^ Energy costs of 27.0 $/MWh for natural gas and 43.3 $/MWh for hydrogen electrolysis, EEX/GASPOOL EEX/ELIX 200 d average price

### Comparison of economic requirements in different UHS options

**Figure 7.**
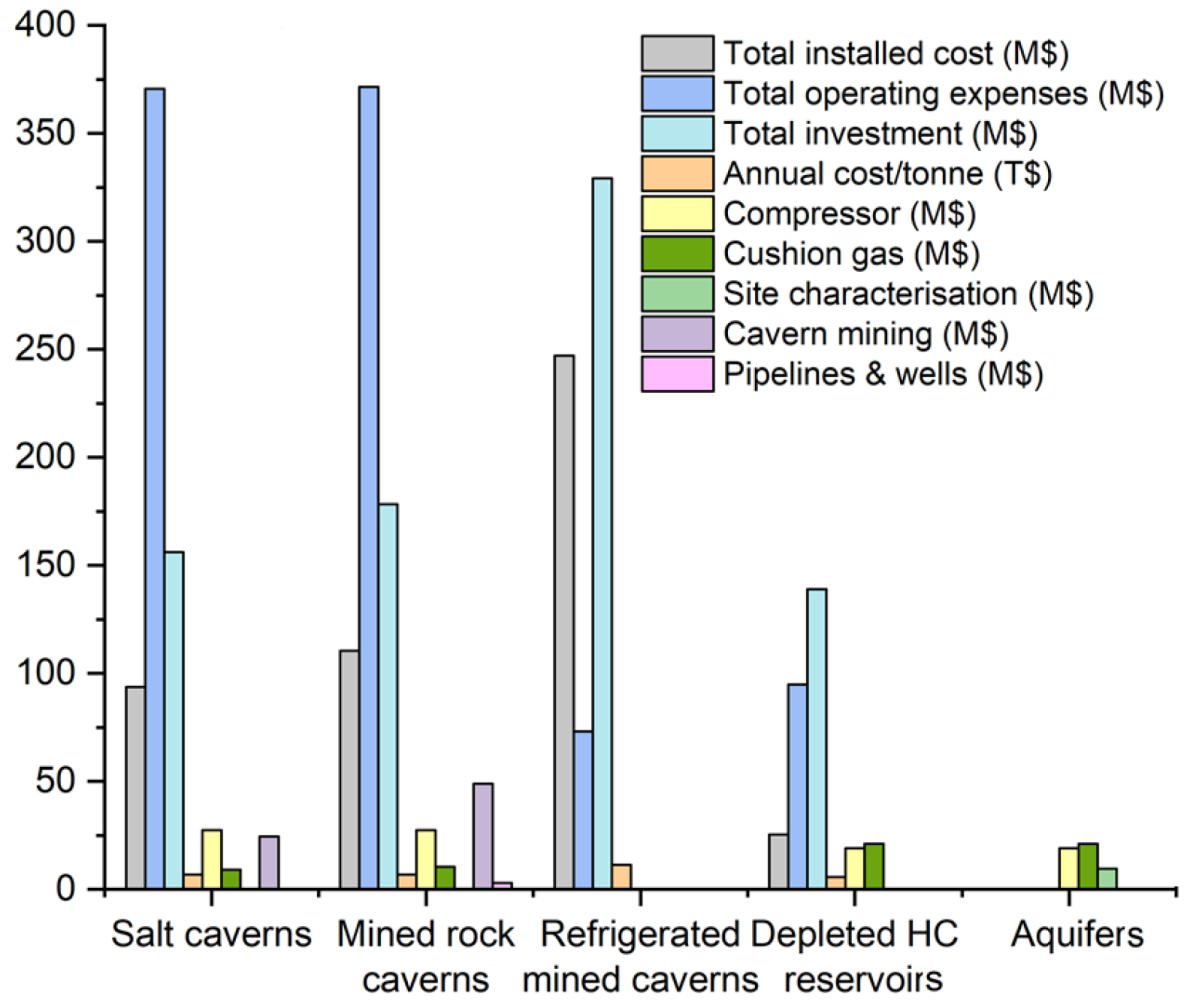
Cost analysis of UHS for different geological formations. A 50 % cushion gas scenario was used for the aquifer option, and an illustrative site characterisation cost of 10 % of the area was used. Columns missing for a total installed cost, total operating expenses, total investment and annual cost/tonne are not estimated for the other options. Columns missing for compressor, cushion gas, site characterisation, cavern mining, and pipelines and wells are not estimated for the refrigerated mined caverns, while their absence in all the other geological formations reflects the negligible cost.

Taking into account the aforementioned needs of every UHS option from an economic perspective, the most attractive method is hydrogen storage in depleted hydrocarbon reservoirs, as there is no need for site characterisation or cavern mining ^44, 45^. Despite the lack of detailed estimations for hydrogen storage in aquifers and based on the capital cost required for the main infrastructure, this option is characterised as another economic technology. When cavern mining is required for final hydrogen storage, the cost increases. Solution mining was more economical than the conventional mining of hard rock caverns. Compared to the regular rock-mined caverns, the limited volume of the refrigerated mined caverns significantly decreases the operating expenses, as shown in Figure 7. The cost of construction of hydrogen storage in aquifers is relatively higher than the depleted hydrocarbon reservoirs, while the lack of a complete estimation of the total installed cost, total operating expenses, and total investment for the storage in aquifers must be highlighted. In the case of depleted hydrocarbon reservoirs, the natural gas reservoirs are more attractive than oil reservoirs, as the residual gas can be exploited as cushion gas ^44^, decreasing the relevant cost. Differences in the depths and volumes of the reservoirs do not influence the overall cost of the construction, even if each cost component differs ^45^. In detail, when the storage takes place in deep depths, a high surface installation for gas compression is required. On the contrary, shallow cavities require a higher construction cost but a lower surface installation.

**Figure 8.**
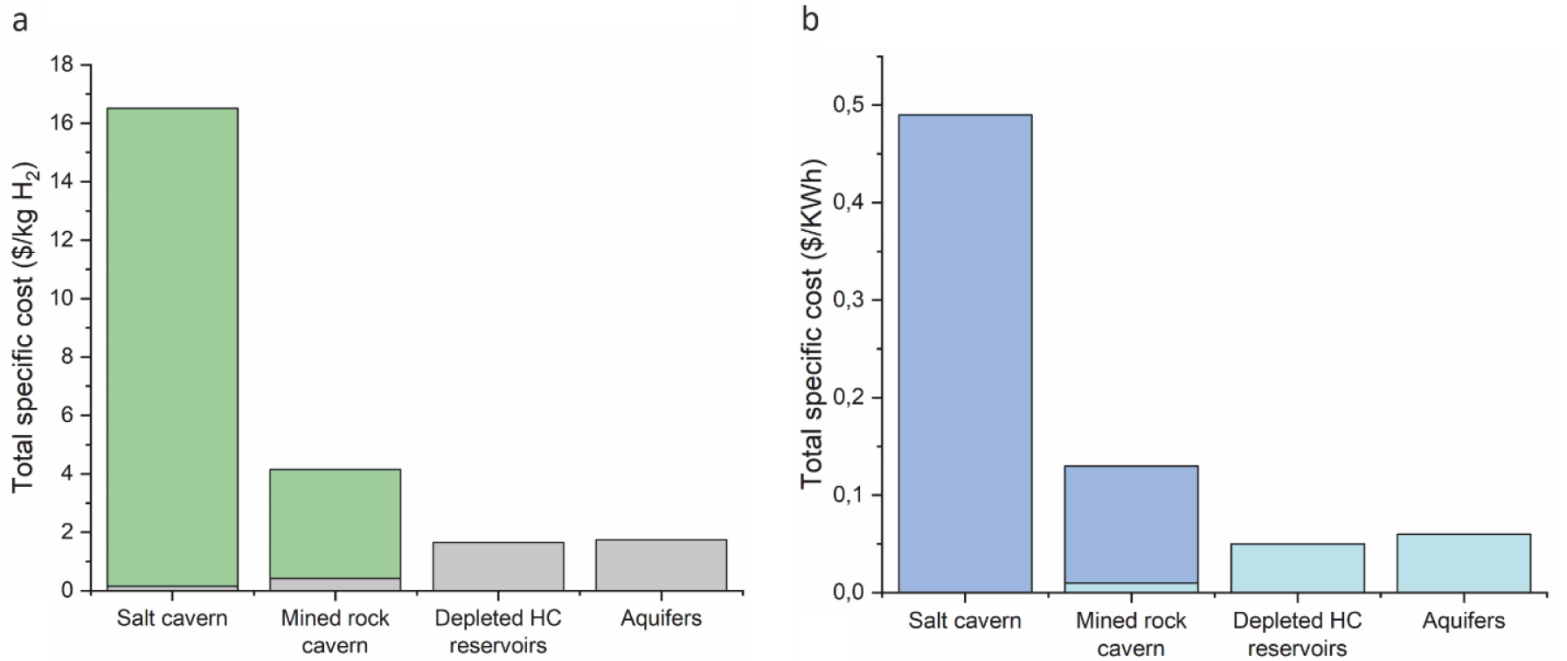
Comparison of total cost estimation for UHS options, considering ( **a**) stored gas and ( **b**) energy. Different colors reflect the cost ranges where they exist.

An estimation of the total cost is given in Figure 8, involving the operation, capital investment and maintenance of hydrogen storage. Critical parameters that can influence the cost variation in specific storage mediums are the cost assumption, the project's components as well as the period between assessment and implementation ^46^. The derived cost given for specific hydrogen production (calculated in kg or KWh) is lower for the case of depleted hydrocarbon reservoirs and aquifers, making them the most affordable options over time. High-cost ranges characterise mined rock caverns and salt caverns. Thus, they can be an affordable option and sometimes an even more financially beneficial option than porous media, depending on the location and the geological storage site properties as well as other significant parameters such as transportation, monitoring, storage, and injection costs ^47^. Potential risks and failures can increase the cost of storage, which can be more easily observed in hydrogen storage in porous rocks. In salt caverns, tightness tests are mandatory for the suitability confirmation of the construction to be ideal for hydrogen storage. Such tests are also increasing the cost.

## Conclusions and outlook

Underground hydrogen storage technologies are still under development. Available cost estimations converted to current prices feature storage in porous media as the most financially affordable option in comparison with the rock cavities. Despite that, one should keep in mind that the literature used in the present review collects data from almost the last forty years. Thus, technological development in the sector could significantly decrease the total cost. For this reason, it was supposed that technological development had the same influence on every UHS option examined, resulting in comparable data.

Until now, depleted hydrocarbon reservoirs and similarly abandoned mines (rock cavities) have been the most optimum options from a financial perspective, as exploitation steps have been omitted due to the already available infrastructure and geological research of the areas in such cases. Apart from the direct financial aspects, considering the green transition and left-behind areas, these options can also leverage local economies. Depleted gas reservoirs prevail due to the presence of remaining gas that sometimes not only does not cause implications but can be used to fulfil the cushion gas requirements, decreasing the cost. In the case of depleted coal mines, belonging to the major category of abandoned mines, the remaining coal could reduce the amount of the withdrawn hydrogen due to the H _2_ adsorption in coals ^30^. This is concluded only by preliminary results, while further investigations are needed. The discussed hydrogen storage methods, offer the potential and the opportunity for upscaling renewable energy solutions and quicker marketing intrusion. This is due to the grid stability solution they offer as complementary to renewable energy.

The exact composition of cushion gas and its reservoir-scale impact must be further examined as it can be a parameter that can influence the cost. Similarly, the dynamics of interfacial interaction between hydrogen and brine that correspond to the significant hydrodynamic challenge in aquifer storage are not tested in detail until now. Moreover, a deep understanding of fluid mixture thermophysical properties and the phase behaviour for specific storage conditions are required for such geological formations. This can be achieved by developing an Equation of States (EOS) to describe in detail the behaviour of hydrogen, brine mixtures, and cushion gas ^48^. This investigation will provide a deep knowledge of the hydrogen purity and especially the processes required for their safe recovery after storage.

Cost analyses for different cycle frequencies require further examination. Data analytics and machine learning tools could be beneficial for advancing geological storage technologies, including site screening, site characterisation, capacity estimation, and risk assessment of every selected site to operational management techniques aiming at detailed analyses and discussions and resulting in cost maintenance. Storage risks must be considered for the final cost analysis of the optimum technology as hydrogen losses may occur due to microbial activity, heterogeneity of the rocks, pore-scale trapping, and dissolution. However, as these processes are site dependent, the optimum underground storage method is strongly influenced by specific site characteristics. Moreover, these risks may be responsible for the presence of environmental impacts. Thus, further investigation is required to connect them with the cost analysis and environmental protection.

## Abbreviations

EU-European Union; ETS-Emissions Trading System; CCUS-Carbon Capture Utilization and Storage; UHS-Underground Hydrogen Storage; LRC-Lined Rock Caverns; HC-Hydrocarbon; US-United States; ROI-Return of Investment; EOS-Equation of States; D-diameter; H-height; $-dollars; €-euros; T$-thousand dollars; M$-million dollars; mD-millidarcy; Mkg-million kilos; MPa-megapascal; st-stere; GWh-gigawatt hours; MWh-megawatt hours; y-year; MMBtu-1million British thermal unit; LH _2_-liquid hydrogen; Nm ^3^- Normal Cubic Meter; ha-hectare; HP-horsepower.

## Ethics and consent

Ethical approval and consent were not required.

## Data Availability

No data are associated with this article.
