# Supplementary material for: Underground hydrogen storage: The techno-economic perspective
Source: Open Res Eur. 2024 May 29;4:17. Originally published 2024 Jan 11. [Version 2] doi: 10.12688/openreseurope.16974.2 (PMC11101923; doi:10.12688/openreseurope.16974.2)
Supplement: Peer review history

# Reviewer report: Reviewer response for version 2

**DOI:** 10.21956/openreseurope.19271.r41046

**Published:** 2024-06-24

**Authors:** Mrityunjay Singh

**Affiliations:** 1 Helmholtz-Centre Potsdam, Potsdam, Germany

Some of the previous comments were not addressed carefully:

 1. How have the authors validated the economic models used in the manuscript? Are there real-world applications or data that corroborate the presented techno-economic analyses?

 2. I am confused for these following two comments: "How do different regulatory frameworks across Europe influence the feasibility of the various underground hydrogen storage options?

**Author response: This is indeed a very good question, however, this paper is concerned with the review and updating the technical and financial data related to such question. Thus, a second paper is prepared on these grounds.**

 What are the long-term viability and potential technological risks associated with each storage method throughout 30 to 50 years?

**Author response: Thank you for your comment. As we described, the technological risks are not part of the purpose of the present review and require further analysis. A reference in the storage risks that need further investigation is given in the conclusions (see lines 521-527).**

 The response to the first question indicates that you are focusing on technical aspects and the response to the 2nd question says that you are not focusing on technical aspects. Please clarify this ambiguity.

 3. Table 6 (originally Table 2): "Table 2: Provide a mathematical formulation to estimate cushion gas amount for different storage formations.

**Author response: The present review does not concern with such deep technical details but rather to inform a potential investor of certain financial data to perform a quick economic analysis. It is envisaged that a reader will follow relevant publications for more technical analysis and information. ** 

 How do you justify this table without a reference or any mathematical formulation? How have you arrived to these numbers?

 4. Suggestions Include sensitivity analyses to test the robustness of the economic models against various economic and technological scenarios. Provide a more detailed assessment of the environmental impacts of each storage technology to complement the techno-economic analysis. Expand the discussion on regulatory challenges and opportunities for deploying hydrogen storage technologies across different jurisdictions. Include a section discussing the steps required to advance the TRLs of less mature hydrogen storage technologies. Provide more detailed breakdowns of cost components, especially hidden costs like maintenance and decommissioning of storage facilities. Add a future outlook section that discusses potential developments in hydrogen storage technologies and their implications. Incorporate case studies or real-world examples of successful implementations of the technologies discussed. Provide a clearer comparative advantage analysis of each technology against traditional and emerging energy storage technologies. Suggest integrating interdisciplinary approaches combining geoscience, economics, and engineering to address complex challenges in hydrogen storage. Offer specific policy recommendations that could facilitate the adoption of underground hydrogen storage technologies. Include a stakeholder analysis to identify key players and their roles in deploying UHS technologies. Discuss potential partnerships or collaborations that could accelerate the development and deployment of UHS technologies. Replace all existing images with clear and high-resolution figures. Provide governing equations to estimate accurate and quantitative parameters related to technical requirements. For salt caverns, list all governing processes and indicate their inter-relation in deciding storage capacity and safety.

**Author response: Thank you for your suggestions. Based on the comments you provided and our responses, we covered some of the topics that were requested. However, as the scope of this review paper is to remain in strict techno-economical characteristics of underground hydrogen storage, we avoided including regulatory or environmental analysis as we believe that they are topics that require deep description and can standalone in future publications.**

 Most of the comments here were not addressed.

Is the review written in accessible language?

Yes

Are all factual statements correct and adequately supported by citations?

No

Are the conclusions drawn appropriate in the context of the current research literature?

Partly

Is the topic of the review discussed comprehensively in the context of the current literature?

Yes

Reviewer Expertise:

Underground hydrogen storage, geological storage of CO2, geothermal energy

I confirm that I have read this submission and believe that I have an appropriate level of expertise to confirm that it is of an acceptable scientific standard, however I have significant reservations, as outlined above.

# Reviewer report: Reviewer response for version 1

**DOI:** 10.21956/openreseurope.18345.r39312

**Published:** 2024-05-17

**Authors:** Mrityunjay Singh

**Affiliations:** 1 Helmholtz-Centre Potsdam, Potsdam, Germany

I have thoroughly reviewed this manuscript titled "Underground Hydrogen Storage: The techno-economic Perspective" by Eleni Gianni et al. The manuscript discusses different underground hydrogen storage (UHS) technologies, assessing their technical and economic feasibility. Here are some critical questions and essential suggestions for improvement that could help enhance the manuscript:

 Questions

 How have the authors validated the economic models used in the manuscript? Are there real-world applications or data that corroborate the presented techno-economic analyses?

 What are the technology readiness levels (TRLs) of the various hydrogen storage methods discussed? How do these levels affect the practical applicability of the findings?

 Is there a comparative analysis of the environmental impacts associated with each hydrogen storage method? Such an analysis could provide a holistic view of the sustainability of each option.

 How do different regulatory frameworks across Europe influence the feasibility of the various underground hydrogen storage options?

 What are the long-term viability and potential technological risks associated with each storage method throughout 30 to 50 years?

 Are the economic assumptions used for the cost analysis sensitive to fluctuations in market conditions, such as changes in the price of hydrogen or the cost of electricity?

 How might geopolitical factors influence the strategic selection of hydrogen storage locations, particularly considering the energy security implications?

 What are the potential impacts of future technological innovations in hydrogen production on the preferred hydrogen storage methods?

 How do the different storage methods ensure the maintenance of hydrogen purity, especially considering the potential for chemical interactions with surrounding materials?

 In Figure 3, it indicates that low-temperature storage has higher volumetric efficiency. However, what is the geomechanical and thermal stress impact due to this very low-temperature gas storage?

 Figure 5: Justify the storage of H2 at a shallower depth of ~200 m. What are the risks associated with this, and what are the economic advantages of this? What is discovery pressure?

 Table 2: Provide a mathematical formulation to estimate cushion gas amount for different storage formations.

 Suggestions

 Include sensitivity analyses to test the robustness of the economic models against various economic and technological scenarios.

 Provide a more detailed assessment of the environmental impacts of each storage technology to complement the techno-economic analysis.

 Expand the discussion on regulatory challenges and opportunities for deploying hydrogen storage technologies across different jurisdictions.

 Include a section discussing the steps required to advance the TRLs of less mature hydrogen storage technologies.

 Provide more detailed breakdowns of cost components, especially hidden costs like maintenance and decommissioning of storage facilities.

 Add a future outlook section that discusses potential developments in hydrogen storage technologies and their implications.

 Incorporate case studies or real-world examples of successful implementations of the technologies discussed.

 Provide a clearer comparative advantage analysis of each technology against traditional and emerging energy storage technologies.

 Suggest integrating interdisciplinary approaches combining geoscience, economics, and engineering to address complex challenges in hydrogen storage.

 Offer specific policy recommendations that could facilitate the adoption of underground hydrogen storage technologies.

 Include a stakeholder analysis to identify key players and their roles in deploying UHS technologies.

 Discuss potential partnerships or collaborations that could accelerate the development and deployment of UHS technologies.

 Replace all existing images with clear and high-resolution figures.

 Provide governing equations to estimate accurate and quantitative parameters related to technical requirements.

 For salt caverns, list all governing processes and indicate their inter-relation in deciding storage capacity and safety.

Is the review written in accessible language?

Yes

Are all factual statements correct and adequately supported by citations?

No

Are the conclusions drawn appropriate in the context of the current research literature?

Partly

Is the topic of the review discussed comprehensively in the context of the current literature?

Yes

Reviewer Expertise:

Underground hydrogen storage, geological storage of CO2, geothermal energy

I confirm that I have read this submission and believe that I have an appropriate level of expertise to confirm that it is of an acceptable scientific standard, however I have significant reservations, as outlined above.

# Response

**Published:** 2024-05-19

**Authors:** Eleni Gianni

**Affiliations:** Centre for Research and Technology Hellas, Greece

How have the authors validated the economic models used in the manuscript? Are there real-world applications or data that corroborate the presented techno-economic analyses? **Response: Thank you for your comment. It allows us to express the process of our work based on data review from other papers. It is assumed that the scientific robustness and hence the validation has been contacted during the peer review of these papers.**

 What are the technology readiness levels (TRLs) of the various hydrogen storage methods discussed? How do these levels affect the practical applicability of the findings?

**Response: Thank you for the comment. It will enhance the quality of our paper. TRL for all these methods varies and as such the resolution analysis offered is quite crude but still offers a basement of discussion. This has been added together with a Table referring to the estimation of TRL and maturity level of technologies (Table 2) in the text for completeness.**

 Is there a comparative analysis of the environmental impacts associated with each hydrogen storage method? Such an analysis could provide a holistic view of the sustainability of each option.

**Response: As the aboveground infrastructure is common for all the investigated technologies, their environmental impact is mainly connected to storage risks. Storage risks are not in the purpose of the present review, however we understand their significance to the cost analysis and the environmental impact and thus we included the following paragraph in the conclusions referring to this topic as potential future work: “Storage risks must be considered for the final cost analysis of the optimum technology as hydrogen losses may occur due to microbial activity, heterogeneity of the rocks, pore-scale trapping, and dissolution. However, as these processes are site dependent, the optimum underground storage method is strongly influenced by specific site characteristics. Moreover, these risks may be responsible for the presence of environmental impacts. Thus, further investigation is required to connect them with the cost analysis and environmental protection”.**

 How do different regulatory frameworks across Europe influence the feasibility of the various underground hydrogen storage options?

**Response: This is indeed a very good question, however, this paper is concerned with the review and updating the technical and financial data related to such question. Thus, a second paper is prepared on these grounds. **  

 What are the long-term viability and potential technological risks associated with each storage method throughout 30 to 50 years?

**Response: Thank you for your comment. As we described, the technological risks are not part of the purpose of the present review and require further analysis. A reference in the storage risks that need further investigation is given in the conclusions (see lines 521-527).**

 Are the economic assumptions used for the cost analysis sensitive to fluctuations in market conditions, such as changes in the price of hydrogen or the cost of electricity? **Response: This is a very important comment. No, the data presented does not take this into account. A sentence stating that has been added to the text (see line 335).**

 How might geopolitical factors influence the strategic selection of hydrogen storage locations, particularly considering the energy security implications?

**Response: Hydrogen storage locations within Europe are of great significance since they provide an energy supply stability in the short and the long term. Furthermore, based on the recent example from the war in Ukraine, they provide an alternative solution for the Green Deal and the energy transition from the traditional fossil fuels and the volatility. Additionally, green hydrogen promotes renewable energy and independence of Europe from alternative sources. (This text has been added to the introduction, lines 56-61). **  

 What are the potential impacts of future technological innovations in hydrogen production on the preferred hydrogen storage methods?

**Response: Thank you for the valuable comment. The discussed hydrogen storage methods, offer the potential and the opportunity for upscaling renewable energy solutions and quicker marketing intrusion. This is due to the grid stability solution they offer as complementary to renewable energy. This was also included in the text (see conclusions).**

 How do the different storage methods ensure the maintenance of hydrogen purity, especially considering the potential for chemical interactions with surrounding materials?

**As it was referred in the conclusions (lines 511-515), this is the current topic of ongoing investigation. However, an addition was performed to give emphasis in the significance of hydrogen purity and its safe recovery (see lines 515-516). **  

 In Figure 3, it indicates that low-temperature storage has higher volumetric efficiency. However, what is the geomechanical and thermal stress impact due to this very low-temperature gas storage?

**Response: Thank you for your comment. This is under current investigation of other researchers (e.g. https://doi.org/10.1016/j.ijhydene.2024.02.189 and https://doi.org/10.1016/j.est.2023.108912). The scope of this paper is to remain in strict techno-economical characteristics of underground hydrogen storage.**

 Figure 5: Justify the storage of H2 at a shallower depth of ~200 m. What are the risks associated with this, and what are the economic advantages of this? What is discovery pressure?

**Response: We would like to thank the reviewer for his comment as based on this we realized the mistake that had been done in Figure 5. The correct depths are 500-2000 m and the picture replaced.**

 Table 2: Provide a mathematical formulation to estimate cushion gas amount for different storage formations.

**Response: The present review does not concern with such deep technical details but rather to inform a potential investor of certain financial data to perform a quick economic analysis. It is envisaged that a reader will follow relevant publications for more technical analysis and information. **  

 Suggestions Include sensitivity analyses to test the robustness of the economic models against various economic and technological scenarios. Provide a more detailed assessment of the environmental impacts of each storage technology to complement the techno-economic analysis. Expand the discussion on regulatory challenges and opportunities for deploying hydrogen storage technologies across different jurisdictions. Include a section discussing the steps required to advance the TRLs of less mature hydrogen storage technologies. Provide more detailed breakdowns of cost components, especially hidden costs like maintenance and decommissioning of storage facilities. Add a future outlook section that discusses potential developments in hydrogen storage technologies and their implications. Incorporate case studies or real-world examples of successful implementations of the technologies discussed. Provide a clearer comparative advantage analysis of each technology against traditional and emerging energy storage technologies. Suggest integrating interdisciplinary approaches combining geoscience, economics, and engineering to address complex challenges in hydrogen storage. Offer specific policy recommendations that could facilitate the adoption of underground hydrogen storage technologies. Include a stakeholder analysis to identify key players and their roles in deploying UHS technologies. Discuss potential partnerships or collaborations that could accelerate the development and deployment of UHS technologies. Replace all existing images with clear and high-resolution figures. Provide governing equations to estimate accurate and quantitative parameters related to technical requirements. For salt caverns, list all governing processes and indicate their inter-relation in deciding storage capacity and safety.

**Response: Thank you for your suggestions. Based on the comments you provided and our responses, we covered some of the topics that were requested. However, as the scope of this review paper is to remain in strict techno-economical characteristics of underground hydrogen storage, we avoided including regulatory or environmental analysis as we believe that they are topics that require deep description and can standalone in future publications.**

# Reviewer report: Reviewer response for version 1

**DOI:** 10.21956/openreseurope.18345.r39313

**Published:** 2024-05-13

**Authors:** Soon Hyeong So

**Affiliations:** 1 Research Institute of Energy and Resources, Research Institute of Advanced Materials, and Department of Materials Science and Engineering,, Seoul National University, Seoul, South Korea

This article is about underground hydrogen storage in terms of technique and economics. Because the period comes when development of renewable energy is highly desired, this article is very timely. Personally, I as a researcher engaged in hydrogen storage based on porous solid materials, feel very interested in this article and read this article with happiness. I believe that this simple and concise article would be acceptable after minor revision.

 1. I hope to correct your English through the whole manuscript. For example, in abstract, it would be better if the authors changed expression from "separated in" to "classified into"

 2. I wish the authors supplemented the introduction part by explaining broad concepts about hydrogen storage not limited to underground hydrogen storage.

 3. I think the beginning part of Technical requirements from 1 to f is difficult to deliver what the authors want to deliver. Please supplement or remove this part for readers' understanding.

Is the review written in accessible language?

Partly

Are all factual statements correct and adequately supported by citations?

Yes

Are the conclusions drawn appropriate in the context of the current research literature?

Yes

Is the topic of the review discussed comprehensively in the context of the current literature?

Yes

Reviewer Expertise:

My specific research area is hydrogen storage based on porous materials.

I confirm that I have read this submission and believe that I have an appropriate level of expertise to confirm that it is of an acceptable scientific standard.

# Reviewer report: Reviewer response for version 1

**DOI:** 10.21956/openreseurope.18345.r39310

**Published:** 2024-05-09

**Authors:** Zaid Jangda

**Affiliations:** 1 Institute of GeoEnergy Engineering, Heriot-Watt University, Edinburgh, Scotland, UK

The article provides a summary of the various options available for UHS and includes an economic comparison of these options. The economic comparison is novel and would help in ascertaining the best option for UHS. However, as acknowledged by the authors, the site-specific nature of the economic comparison may restrict its broader applicability. While the methodology is adaptable, providing a more thorough definition could enhance its utility across diverse sites. While I lack expertise in cost analysis, I note that the inclusion of main outputs within the text could improve readability alongside the provided tables.

 In terms of comparison of the different options for UHS, the details are limited. One of the main differences between cavern and porous media storage is the storage capacity, which is not sufficiently highlighted in the article. Moreover, abandoned mines and depleted reservoirs are different categories and should  be discussed separately. For some storage options, the details are not well defined. I would suggest to provide a high-level overview of each category before providing the technical requirements. The review papers cited in the article are a good source for this information. Technical requirements for rocks caverns are well defined. Other categories can also be detailed similarly.

 In the Introduction section it would help to add the production cost of each colour of hydrogen mentioned in the introduction. The introduction also mentions targets/plans have been set but does not provide those targets/plans. I would also advise to add a comparison of the storage capacity between small-storage (batteries etc), surface storage (tanks etc) and subsurface storage to provide a understanding of the difference in storage capacities (this could be an image).

 The scientific challenges potentially impacting underground hydrogen storage operations are not discussed in the article. These include hydrogen losses due to microbial activates, pore-scale trapping, heterogeneity of the rocks, dissolution (mentioned briefly) etc. Although this may not be the primary focus of the article, addressing these aspects contributes to a holistic understanding of the UHS system. Recent research papers have studied these parameters in detail and should be referenced.  

 Conclusion mentions depleted coal reservoirs for the first time. Does that refer to abandoned mines? This should be clarified.

 Ensuring coherence in sentence structure and rectifying grammatical errors, as well as maintaining consistent tense throughout the article, would enhance readability and overall quality. Specifically the following parts of the article need refinement,

1. The latter part of the abstract
2. 'State of the art' - not clear what this means? - 2nd paragraph
3. Salt caverns - technical requirement
4. Aquifers - technical requirement

Is the review written in accessible language?

Partly

Are all factual statements correct and adequately supported by citations?

Partly

Are the conclusions drawn appropriate in the context of the current research literature?

Yes

Is the topic of the review discussed comprehensively in the context of the current literature?

Partly

Reviewer Expertise:

Pore-scale imaging, hydrogen storage in subsurface rocks.

I confirm that I have read this submission and believe that I have an appropriate level of expertise to confirm that it is of an acceptable scientific standard, however I have significant reservations, as outlined above.

# Response

**Published:** 2024-05-19

**Authors:** Eleni Gianni

**Affiliations:** Centre for Research and Technology Hellas, Greece

The article provides a summary of the various options available for UHS and includes an economic comparison of these options. The economic comparison is novel and would help in ascertaining the best option for UHS. However, as acknowledged by the authors, the site-specific nature of the economic comparison may restrict its broader applicability. While the methodology is adaptable, providing a more thorough definition could enhance its utility across diverse sites. While I lack expertise in cost analysis, I note that the inclusion of main outputs within the text could improve readability alongside the provided tables.

**Response: Thank you for your kind words. Based on all the Reviewers’ comments, we believe our manuscript's quality has been enhanced. The main outputs of the present review has been provided in the last paragraph of the introduction to help the audience's readability.**

 In terms of comparison of the different options for UHS, the details are limited. One of the main differences between cavern and porous media storage is the storage capacity, which is not sufficiently highlighted in the article. Moreover, abandoned mines and depleted reservoirs are different categories and should  be discussed separately. For some storage options, the details are not well defined. I would suggest to provide a high-level overview of each category before providing the technical requirements. The review papers cited in the article are a good source for this information. Technical requirements for rocks caverns are well defined. Other categories can also be detailed similarly.

**Response: A figure (Figure 2) with a comparison of the storage capacities between underground and aboveground facilities has been added to the manuscript. The manuscript clearly states that abandoned mines and depleted hydrocarbon reservoirs belong to different categories, see Lines 109-110. However, as both categories refer to caverns with installed infrastructure, their technical requirements are similar and for the avoidance of repetition, we stated in Lines 130-133: “•          4. Abandoned mines will be described together with depleted hydrocarbon reservoirs belonging to different major categories. In both cases, previous utilization has already developed the main infrastructure.” For a more detailed description of porous media and salt caverns that belong to rock caverns but require a different procedure of development, two new tables (Tables 4 & 5) were added in the main text. **  

 In the Introduction section it would help to add the production cost of each colour of hydrogen mentioned in the introduction. The introduction also mentions targets/plans have been set but does not provide those targets/plans. I would also advise to add a comparison of the storage capacity between small-storage (batteries etc), surface storage (tanks etc) and subsurface storage to provide a understanding of the difference in storage capacities (this could be an image).

**Response: Thank you for your comment. We believe that including the production cost of each colour will enhance the readability of our manuscript. Thus, we included Table 1 with each colour's available production costs. The number of the other Tables was modified accordingly. We believe that our response to your previous comment and the inclusion of a new image fulfills your suggestion to include a comparison of storage capacity between small storage, surface storage and subsurface storage.**

 The scientific challenges potentially impacting underground hydrogen storage operations are not discussed in the article. These include hydrogen losses due to microbial activates, pore-scale trapping, heterogeneity of the rocks, dissolution (mentioned briefly) etc. Although this may not be the primary focus of the article, addressing these aspects contributes to a holistic understanding of the UHS system. Recent research papers have studied these parameters in detail and should be referenced. 

**Response: Thank you for your comment. As you already stated, this is not the primary focus of the article, however, we understand the significance of hydrogen loss in cost analysis and thus, we included the following sentences in the conclusions: “Storage risks must be considered for the final cost analysis of the optimum technology as hydrogen losses may occur due to microbial activity, heterogeneity of the rocks, pore-scale trapping, and dissolution. However, as these processes are site dependent, the optimum underground storage method is strongly influenced by specific site characteristics. Moreover, these risks may be responsible for the presence of environmental impacts. Thus, further investigation is required to connect them with the cost analysis and environmental protection.”**  

 Conclusion mentions depleted coal reservoirs for the first time. Does that refer to abandoned mines? This should be clarified.

**Response: Thank you for your comment! The coal mines were firstly referred in line 291 as category of abandoned mines. However, there is an understandable confusion as they were referred to as coal mines and not depleted coal reservoirs. For this reason, line 291 modified to refer to depleted coal mines and conclusions (line 502) modified accordingly: “In the case of depleted coal mines, belonging to the major category of abandoned mines, the remaining coal could reduce the amount…..”.  **  

 Ensuring coherence in sentence structure and rectifying grammatical errors, as well as maintaining consistent tense throughout the article, would enhance readability and overall quality. Specifically the following parts of the article need refinement,

1. The latter part of the abstract
2. 'State of the art' - not clear what this means? - 2nd paragraph
3. Salt caverns - technical requirement
4. Aquifers - technical requirement

**Response: Thank you for your comment. Several parts have been modified in the manuscript given with track changes to enhance readability and overall quality of the paper.**
